# Chinese Marine Materia Medica

**DOI:** 10.3390/md12010193

**Published:** 2014-01-07

**Authors:** Peter Proksch

**Affiliations:** Institut für Pharmazeutische Biologie, Universität Düsseldorf, Gebäude 26.23, Universitätsstraße 1, 40225 Düsseldorf, Germany; E-Mail: proksch@uni-duesseldorf.de; Tel.: +49-211-8114163; Fax: +49-211-8111923

China is one of the first countries to use marine materia medica for treating diseases. Ancient books on Chinese herbology, such as *Shennong Bencaojing* (*Shennong’s Classic of Materia Medica*), *Xinxiu Bencao* (*Newly Revised Materia Medica*) and *Bencao Gangmu* (*Compendium of Materia Medica*), have detailed more than 110 marine herbs and thousands of marine herbal formulas (including those for Chinese food therapy). A great deal of information on marine herbs and their applications in medicine, collected over thousands of years, has provided an important foundation for modern research in the area of marine drugs. Thanks to these records and references, the research and development of modern Chinese marine drugs continue to evolve and mature. Since the middle of the 20th century, special attention has been paid to traditional Chinese medicine, resulting in a significant increase in the number of newly discovered marine herbs. Comprehensive surveys in the past have also created a wealth of data on the pharmacology, chemistry, biology and ecology of marine medicinal bioresources.

After thousands of years of research, historical references to traditional marine herbs are scattered throughout ancient books, local chronicles, medical books, or monographs on medicinal herbs. Unfortunately, there is no systematic way to collate or scientifically verify these references. Furthermore, during the last century, scientists around the world have accumulated large quantities of information on marine natural products, but these are also scattered throughout academic books and journals.

Professor Huashi Guan, a member of the Chinese Academy of Engineering, is aware of the historical background and importance of marine drugs in modern research. He therefore proposed a national project to survey marine medicinal bioresources in China. A special program in the project, dubbed “Project 908” (Chinese Offshore Investigation and Assessment), was approved by China’s State Council, led by Professor Guan himself, and conducted by the School of Medicine and Pharmacy and the Key Laboratory of Marine Drugs of the Ministry of Education at the Ocean University of China in Qingdao. A systematic and large-scale evaluation of Chinese marine medicinal bioresources, carried out over a period of five years, has now been completed. The results represent an important advance in the comprehensive and systematic understanding of Chinese marine medicinal bioresources.

**Figure 1:**
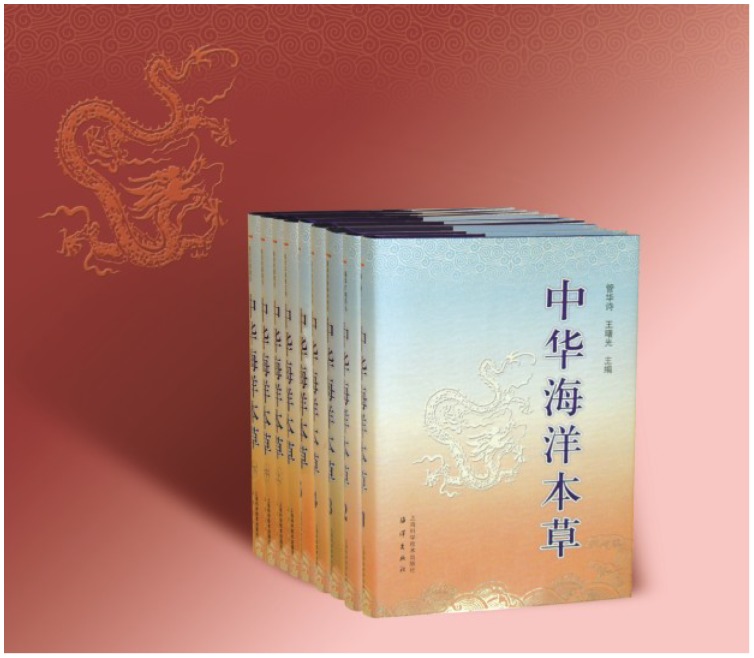
Picture of *Chinese Marine Materia Medica*.

In addition, Professor Guan has compiled a comprehensive encyclopedia entitled *Chinese Marine Materia Medica* (Figure 1). Bringing together the information held in numerous ancient records with modern research findings, the encyclopedia is set to become a classic. The compilation took five years to complete and includes contributions from 300 experts and scholars from more than 40 local universities and research institutions working in various fields, including traditional Chinese medicine, marine biology, microbiology, chemistry and pharmacology. As the first comprehensive reference of its kind, the encyclopedia comprises nine exquisitely bound volumes, contains over 14 million Chinese characters and boasts more than 1500 colored pictures of various marine medicinal organisms and 20,000 chemical structures of marine natural products.

The first five volumes of *Chinese Marine Materia Medica* are the main volumes. The remaining volumes, *Marine Medicinal Microorganisms* (Volume 6) and *Marine Natural Products* (Volumes 7–9), are the supplementary volumes.

The main volumes provide an extensive overview of the history of Chinese marine materia medica, tracing its origin and development. These volumes not only describe the natural habitat, current status, identification, cultivation and harvest of marine organisms with pharmaceutical potential, but also discuss the protection of rare and endangered species. Various sections cover 613 kinds of drugs, 3100 prescriptions, and 1479 marine medicinal organisms, particularly those with potential medicinal values. The work records a wealth of information on traditional Chinese marine medicines, including their scientific names, common names, origin, identification, preparation methods, properties, efficacy, compatibility, usage, dosage, safety, prescription and clinical applications. It provides a systematic record of morphological and ecological features, distribution, collection and storage of marine medicinal organisms, as well as the chemical constituents, pharmacology and toxicology of marine drugs. The editors have also re-examined the scientific names of marine medicinal organisms—particularly those that have been historically mixed up—and made more than 200 corrections. Also, for the very first time, the editors have recorded the fingerprint spectra of 21 important organisms. These records provide an important scientific foundation for the research and development of modern marine drugs and an informative basis for policy makers to make scientific decisions on the use and protection of marine medicinal bioresources.

The supplementary volume *Marine Medicinal Microorganisms* focuses on marine microorganisms, a resource that is relatively unexploited. Marine microorganisms live in extreme environments with high salinity, high pressure, low temperature and low light levels. They have diverse secondary metabolites—often with unusual and novel structures—that have great potential for medical use and are fast becoming a focus in pharmaceutical research. The supplementary volume collates more than 300 marine microorganisms and detailed information on the biology, chemistry and pharmacology of their secondary metabolites.

The three remaining supplementary volumes *Marine Natural Products* are professional references containing extensive data related to marine natural products. Since the beginning of the 20th century, rapid progress has been made in this field. Many extremely active compounds with unique and novel structures have been developed as effective drugs or lead compounds. These supplementary volumes, which include detailed information on the sources, structures, spectroscopic characteristics and biological activities of more than 20,000 marine natural products, can be used as a fast and convenient retrieval index. The text provides full and accurate information for those studying marine drugs and marine herbs.

*Chinese Marine Materia Medica* was compiled on the bases of investigation, excavation and textual research of Chinese traditional marine medicinal bioresources. It will be invaluable to verify and annotate the functions and mechanisms of traditional marine drugs, and more importantly, to analyze and evaluate chemical compositions and biological activities to find new medicinal resources by using modern scientific methods and techniques. Undoubtedly, this will be beneficial to research and develop traditional Chinese marine drugs. *Chinese Marine Materia Medica* is an encyclopedia that records the 3600-year historical development of Chinese marine drugs. It integrates classical theories, such as traditional Chinese medicine, with modern science and technology in diverse areas, such as chemistry, pharmacology and biology. This encyclopedia also represents a milestone in the scientific development of marine drugs, and is the amalgamation of the Eastern and Western ways of thinking. The main and supplementary volumes reinforce as well as complement each other. The work is destined to become a historical legacy and enlightenment for future generations.

